# Supplementary material for: Testing relationship between plant productivity and diversity in a desertified steppe in Northwest China
Source: PeerJ. 2019 Jul 10;7:e7239. doi: 10.7717/peerj.7239 (PMC6626518; doi:10.7717/peerj.7239)
Supplement: Table S1 [file peerj-07-7239-s001.doc]

**Supplementary Table 1** Occurrence frequency of all plant species in desertified steppe

| *Stipa bungeana*  communities | | *Agropyron mongolicum*  communities | | *Glycyrrhiza uralensis*  communities | | *Sophora alopecuroides*  communities | | *Artemisia ordosica*  communities | | *Cynanchum komarovii*  communities | |
| --- | --- | --- | --- | --- | --- | --- | --- | --- | --- | --- | --- |
| Species name | Fi | Species name | Fi | Species name | Fi | Species name | Fi | Species name | Fi | Species name | Fi |
| *Stipa bungeana* | 1.00 | *Agropyron mongolicum* | 1.00 | *Glycyrrhiza uralensis* | 1.00 | *Sophora alopecuroides* | 1.00 | *Artemisia ordosica* | 1.00 | *Cynanchum komarovii* | 1.00 |
| *Potentilla bifurca* | 0.13 | *Lespedeza bicolor* | 1.00 | *Peganum harmala* | 0.47 | *Pennisetum flaccidum* | 1.00 | *Astragalus melilotoides* | 0.67 | *Oxytropis racemosa* | 1.00 |
| [*Artemisia*](../../../../D:/%25E6%259C%2589%25E9%2581%2593/Youdao/Dict/6.3.69.4001/resultui/frame/javascript:void(0)%3B)  [*scoparia*](../../../../D:/%25E6%259C%2589%25E9%2581%2593/Youdao/Dict/6.3.69.4001/resultui/frame/javascript:void(0)%3B) | 0.13 | *Ixeris chinensis* | 0.67 | *Eragrostis pilosa* | 0.37 | *Euphorbia humifusa* | 0.20 | *Agropyron mongolicum* | 1.00 | *Polygala tenuifolia* | 0.87 |
| *Green bristlegrass* | 0.33 | *Heteropappus altaicus* | 1.00 | *Leymus secalinus* | 0.67 | *Green bristlegrass* | 0.13 | *Salsola ruthenica* | 0.67 | *Convolvulus ammannii* | 0.47 |
| *Ixeris chinensis* | 0.13 | *Pennisetum flaccidum* | 0.67 | *Corispermum hyssopifolium* | 0.33 | *Herba ixeris* | 0.07 | *Corispermum hyssopifolium* | 0.40 | *Cleistogenes squarrosa* | 0.33 |
| *Salsola ruthenica* | 0.07 | *Stipa capillata* | 0.33 | *Herba ixeris* | 0.13 | *Salsola ruthenica* | 0.13 | *Heteropappus altaicus* | 0.33 | *Stipa capillata* | 0.33 |
| *Artemisia vestita* | 0.33 | *Salsola ruthenica* | 0.47 | *Salsola ruthenica* | 0.07 | *Artemisia vestita* | 0.07 | *Ixeris chinensis* | 0.17 | *Agropyron mongolicum* | 0.67 |
| *Euphorbia esula* | 0.47 | *Polygala tenuifolia* | 0.67 | *Green bristlegrass* | 0.13 | *Cynanchum thesioides* | 0.13 | *Bassia dasyphylla* | 0.33 | *Euphorbia esula* | 0.57 |
|  |  | *Green bristlegrass* | 0.13 | *Lantern halyard* | 0.20 |  |  | *Cynanchum komarovii* | 0.07 | *Lespedeza bicolor* | 0.17 |
|  |  | *Cleistogenes squarrosa* | 0.07 | *Sophora flavescens* | 0.20 |  |  | *Green bristlegrass* | 0.13 | *Heteropappus altaicus* | 0.07 |
|  |  |  |  | *Euphorbia humifusa* | 0.47 |  |  |  |  | *Salsola collina* | 0.27 |
|  |  |  |  | *Cleistogenes squarrosa* | 0.33 |  |  |  |  | *Artemisia vestita* | 0.37 |
|  |  |  |  | *Tribulus terrestris* | 0.07 |  |  |  |  | *Oxytropis aciphylla* | 0.27 |
|  |  |  |  | *Agropyron mongolicum* | 0.13 |  |  |  |  | *Scorzonera divaricata* | 0.17 |
|  |  |  |  | *Cynanchum komarovii* | 0.47 |  |  |  |  | *Ixeris chinensis* | 0.33 |
