# Supplementary material for: Testing relationship between plant productivity and diversity in a desertified steppe in Northwest China
Source: PeerJ. 2019 Jul 10;7:e7239. doi: 10.7717/peerj.7239 (PMC6626518; doi:10.7717/peerj.7239)
Supplement: Table S2 — SW-soil water content, BD-bulk density, EC-electrical conductivity, pH-pH value, TC-total carbon , TN-total nitrogen, TP-total phosphorus, AP- available phosphorus AN-available nitrogen, SMB-C-soil microbial biomass carbon, SMB-N-soil microbial biomass nitrogen. [file peerj-07-7239-s002.doc]

**Supplementary Table 2** Pearson’s correlation among plant diversity, productivity and soil factors. SW-soil water content, BD-bulk density, EC-electrical conductivity, pH-pH value, TC-total [carbon](../../../../Administrator/AppData/Local/Microsoft/Windows/Users/Administrator/AppData/Local/Yodao/DeskDict/frame/20141120194213/javascript:void(0)%3B), TN-total nitrogen, TP-total phosphorus, AP-available phosphorus, AN-available nitrogen, SMB-C-soil microbial biomass carbon, SMB-N-soil microbial biomass nitrogen.

| Item | Richness  index | Evenness  index | Shannon-Wiener index | Simpson’s  index | AGB | BGB | litters |
| --- | --- | --- | --- | --- | --- | --- | --- |
| BD | -0.226 | -0.606** | -0.407* | -0.576** | -0.182 | -0.242 | -0.321 |
| EC | 0.270 | 0.065 | 0.213 | 0.068 | -0.082 | 0.270 | 0.346 |
| pH | -0.150 | -0.207 | -0.048 | -0.129 | -0.163 | -0.133 | -0.102 |
| SW | 0.146 | 0.490* | -0.294 | 0.437* | 0.516** | 0.490* | 0.088 |
| TC | 0.548** | 0.539** | 0.559** | 0.687** | 0.551** | 0.512** | 0.599** |
| TN | 0.418* | 0.496* | 0.680** | 0.796** | 0.142 | 0.408* | 0.050 |
| TP | 0.312 | 0.608** | 0.641** | 0.777** | 0.226 | 0.311 | 0.309 |
| AP | 0.454* | 0.645** | 0.439* | 0.610** | -0.232 | 0.489* | 0.164 |
| AN | 0.532** | 0.666** | 0.477* | 0.773** | 0.223 | 0.541** | 0.128 |
| SMB-C | 0.552** | 0.715** | 0.689** | 0.785** | 0.000 | -0.088 | 0.364 |
| SMB-N | 0.554** | 0.482* | 0.636** | 0.658** | -0.326 | -0.489 | -0.071 |

** Correlation is significant at the 0.01 level (2-tailed), * correlation is significant at the 0.05 level (2-tailed)
