# Supplementary material for: Testing relationship between plant productivity and diversity in a desertified steppe in Northwest China
Source: PeerJ. 2019 Jul 10;7:e7239. doi: 10.7717/peerj.7239 (PMC6626518; doi:10.7717/peerj.7239)
Supplement: Table S3 — SW-soil water content, BD-bulk density, EC-electrical conductivity, pH-pH value, TC-total carbon, TN-total nitrogen, TP-total phosphorus, AP-available phosphorus, AN-available nitrogen, SMB-C-soil microbial biomass carbon, SMB-N-soil microbial biomass nitrogen. [file peerj-07-7239-s003.doc]

**Supplementary Table 3** Results of redundancy analysis (RDA) using forward selection with the Monte Carlo permutation test. Values are for Axes 1 and 2 plotted in the RDA diagram. SW-soil water content, BD-bulk density, EC-electrical conductivity, pH-pH value, TC-total [carbon](../../../../Administrator/AppData/Local/Microsoft/Windows/Users/Administrator/AppData/Local/Yodao/DeskDict/frame/20141120194213/javascript:void(0)%3B), TN-total nitrogen, TP-total phosphorus, AP-available phosphorus, AN-available nitrogen, SMB-C-soil microbial biomass carbon, SMB-N-soil microbial biomass nitrogen

| Axis | Axis1 | Axis2 |
| --- | --- | --- |
| Eigenvalues | 0.712 | 0.189 |
| Cumulative percentage variance | - | - |
| Species date | 71.2 | 89.1 |
| Species-environment relationship | 71.2 | 89.1 |
| Summary of Monte Carlo test： | - | - |
| *p*-value | 0.001** | 0.005** |
| *F*-ratio | 5.12 | 3.07 |
| Species-environment correlation | 1.000 | 1.000 |
| Variation explained (%) | 98.03 | - |
| Sum of all eigenvalues | 1.000 | - |
| Sum of all canonical eigenvalues | 1.000 | - |
| BD | -0.856** | -0.765** |
| EC | 0.523* | 0.302 |
| pH | -0.903** | -0.639** |
| SW | 0.714** | 0.517* |
| TC | 0.923** | 0.526* |
| TN | 0.712** | 0.352 |
| TP | 0.136 | 0.285 |
| AP | 0.429 | 0.089 |
| AN | 0.125 | 0.503* |
| SMB-C | 0.855** | 0.763** |
| SMB-N | 0.623** | 0.742** |

**p*<0.05，***p*<0.01
